# A Case of Type 2 Sialidosis With Deletion of a Single Nucleotide at Position c.947 of the Neuraminidase 1 (NEU1) Gene

**DOI:** 10.7759/cureus.20389

**Published:** 2021-12-13

**Authors:** Moath Hassan, Mohammed A Alharbi, Reem Y Alhassani, ARWA A Hussain, Ramziyyah Y Kamfar

**Affiliations:** 1 Pediatrics, Maternity and Children's Hospital, Makkah, SAU; 2 Medicine, Maternity and Children's Hospital, Makkah, SAU; 3 Medicine, Umm Al-Qura University, Makkah, SAU; 4 Pediatrics, Maternity and Children’s Hospital, Makkah, SAU

**Keywords:** pediatric, sialidosis type ii, polypheny, neu1, common mutation

## Abstract

Sialidosis is a rare, autosomal recessive inherited disorder caused by α-N-acetyl neuraminidase deficiency resulting from a mutation in the neuraminidase gene (NEU1), located on 6p21.33. A definitive diagnosis is made after the identification of a mutation in the NEU1 gene. An association exists between the impact of the individual mutations and the severity of the clinical presentation of sialidosis. Despite being uncommon, sialidosis has enormous clinical relevance due to its debilitating character. A complete understanding of the underlying pathology remains a challenge, which in turn limits the development of effective therapeutic strategies. We present a case of diagnosed congenital sialidosis type II.

## Introduction

Sialidosis is a rare autosomal recessive disorder characterised by progressive lysosomal storage of sialylated glycopeptides and oligosaccharides caused by a deficiency of the enzyme neuraminidase [1]. The human neuraminidase gene is located at chromosome band 6p21.33. Until 1977, deficiency of neuraminidase 1 (NEU1) was thought to be associated with classical mucolipidosis [2,3]. In 1977, the term sialidosis was first used to describe the syndrome of two siblings having a visual impairment and mild neurological manifestations. Enzymatic assays in cultured fibroblasts and leukocytes from these siblings exhibited an isolated deficiency of NEU1 [2,3].

Sialidosis was classified into two types: type I is mild (normosomatic), and type II is referred to as cherry-red spot myoclonus syndrome. Sialidosis type II is the severe and earlier onset (dysmorphic). Type II is subdivided into congenital, infantile, and juvenile forms. The congenital form is associated with either hydrops fetalis and stillbirth or neonatal ascites and death at an early age. Its features include facial oedema, inguinal hernias, hepatosplenomegaly, stippling of the epiphyses, and periosteal cloaking [3]. We report an infant diagnosed with congenital sialidosis type II.

## Case presentation

A two-month-old preterm girl (37 weeks) was admitted to the newborn intensive care unit (NICU) for one week as a case of respiratory distress and septic shock. In the follow-up visit, the patient came with abdominal distension. The abdominal distention was progressive and had been noticed in the past 2 to 3 weeks. The swelling was limited to the abdomen. No vomiting, jaundice, change in bowel habits (passing stool), melena, or hematemesis was observed. There was no cyanosis or sweating during feeding, no urinary changes, no fever, decreased oral intake, or activity. There was a normal level of consciousness. Furthermore, there was no shortening of breath, cough, or history of contact with a sick patient.

Other systematic assessments were unremarkable. The patient was previously admitted to the hospital at age one month as a case of neonatal septic shock treated with antibiotic treatment.

The mother was infected with COVID-19 during pregnancy, and she was treated conservatively at home. Our patient was the first child in the family, positive consanguinity between parents (first-degree relative) but no family history of inherited diseases, diagnosed syndromes, or stillbirth.

**Figure 1:**
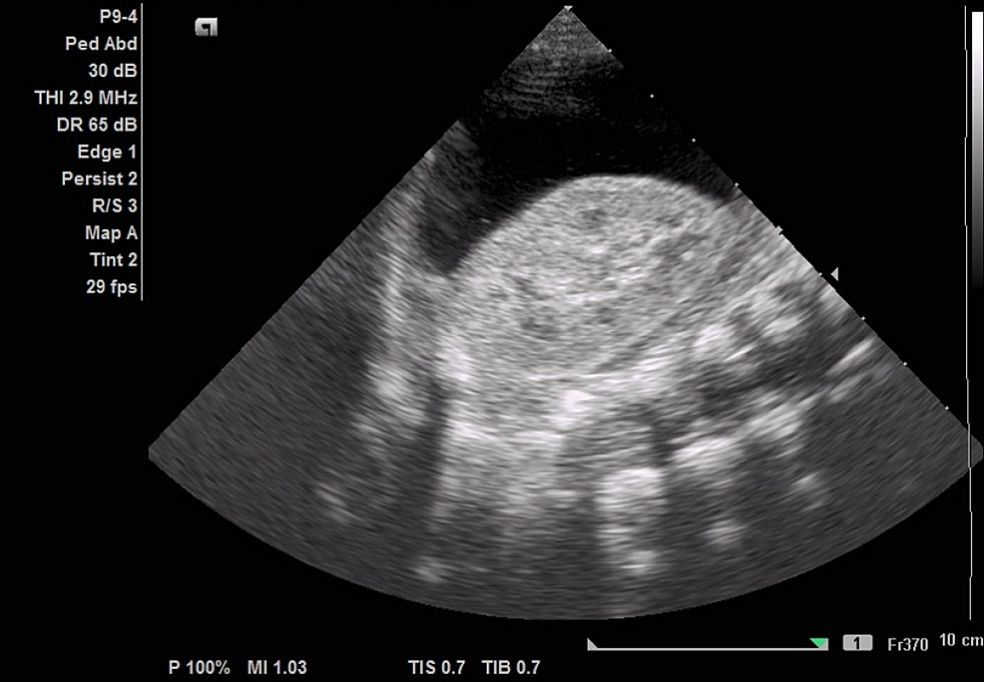
Ultrasound abdomen. Marked ascites with septations is seen.

On examination, the patient looks dysmorphic large forehead, puffy eyes, rounded chubby nose, long philtrum, micrognathia, bell-shaped chest, widely spaced nipple, and sacral dimple). The infant’s growth parameters were as follows: weight: 3.5 kg (15^th^ percentile). Head circumference was as follows: 39 cm (current, 15^th^ centile). Abdominal examination revealed moderate distention of the abdomen, visible veins, and hepatomegaly 2 to 3 cm below the costal margin, no splenomegaly. Cardiovascular examination revealed fine-grade 2 to 3 systolic murmur. No lower limb oedema was noticed. The rest of her physical examination was unremarkable. The ophthalmology assessment showed cloudy corneas. Initial laboratory investigation was within the normal range, except for moderate neutropenia at 670/mm. Renal and liver panels were within the normal range. The urine collected for creatinine and protein results were below the nephrotic range. The patient was admitted as a case of abdominal distention for investigation. Broad-spectrum antibiotics were initiated. X-ray and ultrasound abdomen was performed, and the X-ray was insignificant. Ultrasound showed marked ascites with bilateral plural effusion more on the left side (Figure 1).

**Figure 2:**
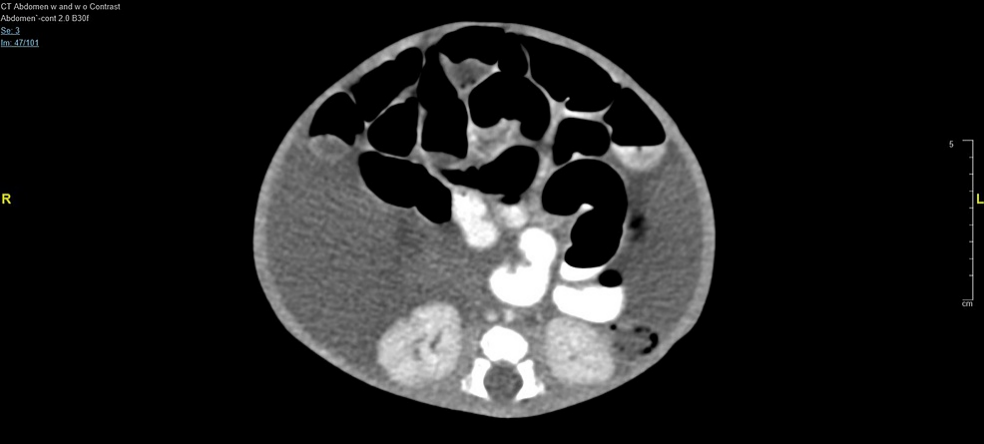
Computed tomography axial view at the level of lumber spine, showed marked abdominopelvic ascites.

Computed tomography (CT) showed marked abdominopelvic ascites (Figure 2).

An echocardiogram showed mild mitral regurgitation, with mildly dilated left ventricle (LV) and LV EF at 51%. Echocardiogram images are not documented or saved anywhere. 

During patient hospitalization, patient symptoms and distention worsened, haemoglobin gradually decreased, and liver and renal markers fluctuated.

Abdominal paracentesis appeared dark yellow, the culture showed no growth, and the alpha-fetoprotein (AFP) was negative. The SAAG calculation (serum ascitic albumin) was <1.1, the creatinine to albumin ratio was repeatedly tested, and the last time was 256 mg/mmol.

The patient was initially managed with congenital nephrotic syndrome, much improvement in patient symptoms and abdominal distention was noticed on albumin therapy and furosemide.

**Figure 3:**
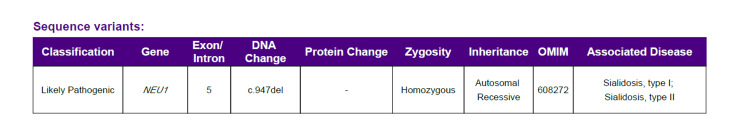
Whole Exome Sequencing test (WES) result

The whole-exome sequencing study result came before discharging the patient and showed NEU1 c.947del (Figure 3), resulting in the deletion of a single nucleotide at position c.947 of the NEU1 gene, causing a frameshift in the protein reading frame. This variant has not been reported in the literature as causative of disease to our knowledge but is expected to cause disease. This is genetically abnormal, and clinical findings suggest this patient has type 2 sialidosis.

## Discussion

We describe a case recognised and diagnosed at an early age as sialidosis type 2. The average age of recognition of sialidosis type 2 is one year and nine months, and the average age of diagnosis is six-and-a-half years [4].

The development of significant renal disease appears to be limited to children with congenital and infantile forms of sialidosis. Nephrotic changes were described in Clifford’s study, which showed IgM, C3, and deposits focal segmental glomerulosclerosis [5]; these pathological findings may be associated with our patient. King et al. described different types of lysosomal storage disease, such as infantile sialidosis, Salla disease, GM1 gangliosidosis, and Gaucher disease associated with ascites [6]. A missense pathogenic variation in exon 4 of the NEU1 gene (c.679G > A; p.Gly227Arg) was identified in all seven patients reviewed in Arora and Setia’s study [4]. In our case, deletion of a single nucleotide at position c.947 of the NEU1 gene was detected, causing a frameshift in the protein reading frame; this variant has not been reported in the literature as causative of disease to our knowledge [7].

In Saudi Arabia, Khalid and Abdul Haleem reported a case of sialidosis but in a stillborn, severely hydropic baby. The diagnosis was made by cultured fibroblast and urine examination [8]. So, our case could be the first case documented in Saudi Arabia.

## Conclusions

Sialidosis is a rare autosomal recessive disorder caused by a deficiency of the enzyme neuraminidase. There are two types of sialidosis: type I and type II, which are subdivided into congenital, infantile, and juvenile forms. We report an infant diagnosed with congenital sialidosis type II at two months of age that presented mainly with abdominal distention and was later found to be accompanied by proteinuria. Through investigation, the whole-exome sequencing study result showed NEU1 c.947del, a variant resulting in the deletion of a single nucleotide at position c.947 of the NEU1 gene, causing a frameshift in the protein reading frame. A complete understanding of the underlying pathology remains a challenge, which limits the development of effective therapeutic strategies. From our literature review, our case could be the first documentation of sialidosis type 2 in Saudi Arabia.
